# Supplementary material for: Nutrigenomic Effects of White Rice and Brown Rice on the Pathogenesis of Metabolic Disorders in a Fruit Fly Model
Source: Molecules. 2023 Jan 5;28(2):532. doi: 10.3390/molecules28020532 (PMC9865196; doi:10.3390/molecules28020532)
Supplement: Supplementary file 1 [file molecules-28-00532-s001.zip › molecules-1921726-supplementary.pdf]

Table S1. Body weight changes and negative geotaxis activity for male and female flies after 7 days of diet intervention.

	RICE CULTIVARS	WEIGHT CHANGES (mg)		NEGATIVE GEOTAXIS (No of flies/10 s)	
**S/N**		**MALE**	**FEMALE**	**MALE**	**FEMALE**
**1**	NCD	4.80±0.10^{a}	9.40±0.36^{b}	9.67±0.08^{a}	9.33±0.28^{a}
**2**	HFD	12.40±0.46^{a}	13.20±0.30^{b}	6.33±0.58^{a}	4.67±0.56^{b}
**3**	AKM_B	4.23±0.15^{a}	8.53±0.12^{b}	9.33±0.35^{a}	8.67±0.18^{b}
**4**	AKM_W	8.50±0.30^{a}	11.77±0.25^{b}	7.03±0.05^{a}	6.33±0.50^{b}
**5**	BAI_B	6.17±0.31^{a}	9.93±0.15^{b}	8.67±0.53^{a}	8.33±0.53^{a}
**6**	BAI_W	10.50±0.30^{a}	11.20±0.30^{b}	7.00±0.00^{a}	6.00±0.00^{b}
**7**	BAB_B	4.97±0.21^{a}	10.10±0.35^{b}	8.67±0.03^{a}	8.00±0.00^{b}
**8**	BAB_W	10.13±0.21^{a}	12.57±0.21^{b}	7.13±0.25^{a}	6.00±0.00^{b}
**9**	DAN_B	4.80±0.10^{a}	10.00±0.36^{b}	9.33±0.45^{a}	8.67±0.50^{b}
**10**	DAN_W	9.07±0.42^{a}	12.57±0.21^{b}	7.23±0.08^{a}	6.33±0.45^{b}
**11**	JEEP_B	5.97±0.55^{a}	9.90±0.20^{b}	9.00±0.00^{a}	8.33±0.58^{b}
**12**	JEEP_W	11.17±0.85^{a}	12.97±0.21^{b}	7.67±0.28^{a}	6.33±0.36^{b}
**13**	JAM_B	5.77±0.06^{a}	8.50±0.00^{b}	8.53±0.50^{a}	8.33±0.58^{a}
**14**	JAM_W	11.40±0.00^{a}	11.13±0.06^{b}	7.33±0.51^{a}	6.67±0.51^{b}
**15**	MBC_B	5.03±0.12^{a}	8.67±0.06^{b}	9.00±0.00^{a}	9.00±1.00^{a}
**16**	MBC_W	9.47±0.12^{a}	12.63±0.06^{b}	6.73±0.54^{a}	5.67±0.49^{b}
**17**	YARW_B	6.00±0.00^{a}	8.80±0.17^{b}	8.67±0.48^{a}	9.67±0.53^{b}
**18**	YARW_W	9.87±0.06^{a}	12.97±0.12^{b}	6.33±0.57^{a}	5.67±1.15^{b}
**19**	KWA_B	4.30±0.00^{a}	8.30±0.00^{b}	9.33±0.39^{a}	9.33±0.54^{a}
**20**	KWA_W	8.83±0.06^{a}	11.83±0.06^{b}	7.33±0.62^{a}	6.47±0.57^{b}
**21**	YARK_B	5.43±0.12^{a}	8.80±0.10^{b}	8.00±0.00^{a}	8.67±1.15^{b}
**22**	YARK_W	9.77±0.06^{a}	12.77±0.06^{b}	6.67±0.40^{a}	6.33±0.51^{a}
**23**	DANB_B	5.57±0.23^{a}	9.13±0.23^{b}	8.33±0.55^{a}	9.33±0.29^{b}
**24**	DANB_W	12.10±0.10^{a}	13.13±0.12^{b}	7.33±0.48^{a}	5.33±0.52^{b}
**25**	BAD_B	4.37±0.32^{a}	9.27±0.06^{b}	8.67±0.33^{a}	8.33±0.47^{a}
**26**	BAD_W	12.27±0.31^{a}	14.20±0.10^{b}	3.33±0.52^{a}	5.00±0.00^{b}
**27**	FARO_B	6.07±0.06^{a}	9.47±0.42^{b}	9.00±0.00^{a}	8.33±0.58^{b}
**28**	FARO_W	11.50±0.00^{a}	12.03±0.06^{b}	5.33±0.25^{a}	6.36±0.43^{b}
**29**	JIR_B	6.10±0.00^{a}	8.80±0.00^{b}	8.00±0.00^{a}	9.00±0.00^{b}
**30**	JIR_W	11.47±0.06^{a}	13.57±0.40^{b}	7.67±0.58^{a}	6.00±0.00^{b}
**31**	YARM_B	5.53±0.64^{a}	9.00±0.00^{b}	9.00±0.00^{a}	8.33±0.50^{b}
**32**	YARM_W	11.20±0.00^{a}	13.50±0.00^{b}	6.70±0.02^{a}	5.00±0.00^{b}

Mean ±SD values between male and female denoted by the same letters in superscript are not significantly different ($p > 0.05$) using two-way ANOVA, multiple comparison was done using *Bonferroni's* test. _B= Brown rice, _W= White rice, (n=30). NCD= Normal diet, HFD= High fat diet *Akai maza hajj* =AKM, *Baingila* =BAI, *Baburashi* =BAB, *Jamila* =JAM, *Maibakincarki* =MBC, *Yarwasagi* =YARW, *Kwandala* =KWA, *Yarkatabore* =YARK, *Danboto* =DANB, *Bakindanboto* =BAD, *Faro 44* =FARO, *Jirkita* =JIR, *Yarkukuma* =YARM, *Dankaushi* =DAN and *Jeep* =JEEP.

Table S2. Effects of rice cultivars on the levels of fasting glucose, trehalose and glycogen for both male and female flies after 7 days of intervention diets.

	INTERVENTION DIETS	GLUCOSE (mg/dL)		TREHALOSE (mg/g sample)		GLYCOGEN (mg/g sample)	
		MALE	FEMALE	MALE	FEMALE	MALE	FEMALE
1	NCD	8.98±1.47^{a}	10.21±1.05^{b}	0.11±0.00^{a}	0.11±0.00^{a}	0.20±0.01^{a}	0.20±0.05^{a}
2	HFD	11.18±1.25^{a}	13.01±0.83^{b}	0.16±0.01^{a}	0.17±0.03^{a}	0.09±0.00^{a}	0.08±0.00^{a}
3	AKM_B	8.61±0.64^{a}	9.80±0.79^{b}	0.09±0.05^{a}	0.10±0.00^{a}	0.31±0.05^{a}	0.20±0.04^{b}
4	AKM_W	10.77±0.16^{a}	12.60±0.29^{b}	0.15±0.06^{a}	0.16±0.04^{a}	0.12±0.03^{a}	0.10±0.01^{a}
5	BAI_B	8.91±0.44^{a}	10.43±1.43^{b}	0.11±0.00^{a}	0.09±0.01^{a}	0.20±0.08^{a}	0.19±0.02^{a}
6	BAI_W	11.86±0.62^{a}	12.53±0.34^{b}	0.17±0.02^{a}	0.17±0.02^{a}	0.10±0.01^{a}	0.09±0.00^{a}
7	BAB_B	8.37±1.03^{a}	10.24±0.35^{b}	0.11±0.00^{a}	0.10±0.01^{a}	0.18±0.04^{a}	0.18±0.04^{a}
8	BAB_W	11.46±0.27^{a}	12.64±0.34^{b}	0.17±0.00^{a}	0.17±0.03^{a}	0.09±0.00^{a}	0.08±0.00^{a}
9	DAN_B	9.07±1.27^{a}	10.21±0.25^{b}	0.10±0.01^{a}	0.11±0.01^{a}	0.20±0.07^{a}	0.20±0.05^{a}
10	DAN_W	11.57±0.44^{a}	12.51±0.21^{b}	0.14±0.03^{a}	0.15±0.04^{a}	0.11±0.01^{a}	0.11±0.00^{a}
11	JEEP_B	9.24±0.20^{a}	11.04±0.60^{b}	0.11±0.01^{a}	0.11±0.01^{a}	0.17±0.02^{a}	0.26±0.06^{b}
12	JEEP_W	11.54±0.14^{a}	12.07±0.56^{a}	0.18±0.05^{a}	0.19±0.05^{a}	0.08±0.00^{a}	0.09±0.00^{a}
13	JAM_B	9.15±0.78^{a}	10.87±0.83^{b}	0.09±0.01^{a}	0.10±0.01^{a}	0.17±0.03^{a}	0.17±0.06^{a}
14	JAM_W	11.87±0.75^{a}	13.10±0.33^{b}	0.12±0.03^{a}	0.13±0.03^{a}	0.09±0.01^{a}	0.08±0.01^{a}
15	MBC_B	8.37±0.49^{a}	8.70±0.77^{a}	0.10±0.02^{a}	0.10±0.02^{a}	0.29±0.02^{a}	0.21±0.02^{b}
16	MBC_W	10.56±0.30^{a}	12.69±0.88^{b}	0.13±0.01^{a}	0.13±0.01^{a}	0.13±0.01^{a}	0.10±0.01^{b}
17	YARW_B	9.21±0.21^{a}	10.33±0.37^{b}	0.11±0.01^{a}	0.11±0.01^{a}	0.28±0.05^{a}	0.20±0.02^{b}
18	YARW_W	12.13±0.33^{a}	12.60±0.17^{a}	0.13±0.03^{a}	0.15±0.05^{a}	0.11±0.01^{a}	0.09±0.01^{a}
19	KWA_B	8.81±0.20^{a}	10.74±0.23^{b}	0.09±0.01^{a}	0.11±0.01^{a}	0.19±0.04^{a}	0.17±0.03^{a}
20	KWA_W	12.15±0.36^{a}	12.80±0.23^{b}	0.13±0.01^{a}	0.16±0.02^{b}	0.11±0.01^{a}	0.08±0.01^{b}
21	YARK_B	9.34±0.39^{a}	10.98±0.11^{b}	0.09±0.02^{a}	0.11±0.01^{a}	0.17±0.03^{a}	0.19±0.04^{a}
22	YARK_W	13.93±0.20^{a}	14.53±0.13^{b}	0.16±0.02^{a}	0.16±0.07^{a}	0.11±0.01^{a}	0.09±0.01^{a}
23	DANB_B	7.92±0.41^{a}	8.22±0.80^{a}	0.09±0.00^{a}	0.10±0.00^{a}	0.30±0.06^{a}	0.24±0.02^{b}
24	DANB_W	13.61±1.09^{a}	14.46±1.57^{b}	0.16±0.04^{a}	0.17±0.05^{a}	0.08±0.00^{a}	0.09±0.00^{a}
25	BAD_B	7.43±0.71^{a}	8.25±0.85^{b}	0.11±0.01^{a}	0.11±0.00^{a}	0.28±0.05^{a}	0.21±0.07^{b}
26	BAD_W	11.57±0.46^{a}	12.96±0.08^{b}	0.18±0.06^{a}	0.19±0.06^{a}	0.12±0.01^{a}	0.07±0.02^{b}
27	FARO_B	9.08±0.36^{a}	8.63±0.97^{a}	0.09±0.01^{a}	0.09±0.01^{a}	0.21±0.02^{a}	0.19±0.03^{a}
28	FARO_W	12.10±0.97^{a}	12.25±0.7^{a}	0.18±0.01^{a}	0.19±0.04^{a}	0.12±0.00^{a}	0.11±0.00^{a}
29	JIR_B	9.13±0.44^{a}	9.22±0.86^{a}	0.11±0.00^{a}	0.11±0.00^{a}	0.20±0.04^{a}	0.17±0.02^{b}
30	JIR_W	12.44±0.99^{a}	12.33±0.79^{a}	0.16±0.02^{a}	0.19±0.06^{b}	0.11±0.00^{a}	0.10±0.00^{a}
31	YARM_B	8.80±0.45^{a}	9.05±0.66^{a}	0.09±0.00^{a}	0.09±0.01^{a}	0.17±0.03^{a}	0.20±0.05^{b}
32	YARM_W	12.26±0.39^{a}	12.80±0.83^{a}	0.20±0.00^{a}	0.20±0.04^{a}	0.08±0.00^{a}	0.08±0.00^{a}

Mean ±SD values between male and female denoted by the same letters in superscript are not significantly different ($p > 0.05$) using two-way ANOVA, multiple comparison was done using *Bonferroni's* test. _B= Brown rice, _W= White rice, (n=30). NCD= Normal diet, HFD= High fat diet *Akai maza hajj* =AKM, *Baingila* =BAI, *Baburashi* =BAB, *Jamila* =JAM, *Maibakincarki* =MBC, *Yarwasagi* =YARW, *Kwandala* =KWA, *Yarkatabore* =YARK, *Danboto* =DANB, *Bakindanboto* =BAD, *Faro 44* =FARO, *Jirkita* =JIR, *Yarkukuma* =YARM, *Dankaushi* =DAN and *Jeep* =JEEP.

Table S3. Effects of rice cultivars on triglyceride level and oxidative stress markers in male and female flies after 7 days of intervention diets.

S/N	INTERVENTION DIETS	TG (mg/dL)		SOD (U/mL)		CAT (U/mL)		MDA (nmol/g)	
		MALE	FEMALE	MALE	FEMALE	MALE	FEMALE	MALE	FEMALE
1	NCD	23.64±3.47^{a}	35.77±8.08^{b}	9.98±0.47^{a}	8.85±0.54^{b}	2.94±0.79^{a}	1.89±0.48^{b}	0.17±0.04^{a}	0.17±0.01^{a}
2	HFD	36.85±9.66^{a}	52.95±14.18^{b}	5.13±0.24^{b}	4.97±0.13^{a}	1.13±0.39^{a}	0.91±0.09^{a}	0.31±0.06^{a}	0.31±0.07^{a}
3	AKM_B	23.26±2.21^{a}	36.82±12.08^{b}	10.01±0.99^{a}	9.72±0.39^{a}	3.16±0.04^{a}	2.26±0.03^{b}	0.16±0.02^{a}	0.18±0.01^{a}
4	AKM_W	36.90±1.65^{a}	44.50±12.49^{b}	5.78±0.15^{a}	5.29±0.37^{a}	1.48±0.39^{a}	1.06±0.00^{b}	0.30±0.03^{a}	0.37±0.02^{b}
5	BAI_B	24.31±6.02^{a}	35.74±14.17^{b}	7.82±0.38^{a}	8.59±0.46^{b}	2.81±0.39^{a}	1.58±0.39^{b}	0.16±0.01^{a}	0.19±0.03^{b}
6	BAI_W	35.79±10.43^{a}	46.87±11.73^{b}	5.37±0.25^{a}	5.11±0.52^{a}	1.13±0.39^{a}	1.03±0.08^{a}	0.32±0.02^{a}	0.33±0.07^{a}
7	BAB_B	24.21±2.03^{a}	37.41±14.39^{b}	7.19±0.35^{a}	8.54±1.61^{b}	2.53±0.03^{a}	1.76±0.06^{b}	0.19±0.00^{a}	0.19±0.01^{a}
8	BAB_W	32.51±9.72^{a}	78.33±9.61^{b}	5.29±0.14^{a}	5.05±0.28^{a}	1.13±0.04^{a}	0.91±0.07^{a}	0.34±0.03^{a}	0.36±0.02^{a}
9	DAN_B	25.09±0.36^{a}	46.23±4.29^{b}	10.39±0.63^{a}	8.15±0.51^{b}	2.94±0.78^{a}	2.03±0.36^{b}	0.15±0.01^{a}	0.15±0.01^{a}
10	DAN_W	36.78±3.03^{a}	52.59±10.35^{b}	5.79±0.30^{a}	5.29±0.37^{a}	1.36±0.17^{a}	1.11±0.30^{a}	0.29±0.04^{a}	0.35±0.04^{b}
11	JEEP_B	25.28±4.19^{a}	36.10±6.84^{b}	7.55±1.41^{a}	7.51±1.79^{a}	2.26±0.14^{a}	2.03±0.35^{a}	0.19±0.00^{a}	0.20±0.01^{a}
12	JEEP_W	35.54±10.70^{a}	53.08±6.43^{b}	5.13±0.24^{a}	4.97±0.13^{a}	0.90±0.08^{a}	0.91±0.39^{a}	0.31±0.03^{a}	0.38±0.06^{b}
13	JAM_B	26.07±3.51^{a}	35.90±2.59^{b}	7.61±0.20^{a}	7.55±0.15^{a}	2.32±0.08^{a}	2.18±0.12^{a}	0.17±0.02^{a}	0.16±0.01^{a}
14	JAM_W	37.30±1.57^{a}	53.02±0.63^{b}	4.96±0.19^{a}	5.01±0.12^{a}	1.04±0.01^{a}	1.14±0.04^{a}	0.28±0.01^{a}	0.36±0.08^{b}
15	MBC_B	24.87±2.42^{a}	29.41±0.52^{a}	12.98±0.46^{a}	12.11±0.62^{b}	3.41±0.16^{a}	3.06±0.09^{b}	0.15±0.04^{a}	0.18±0.01^{b}
16	MBC_W	29.34±2.09^{a}	44.47±2.45^{b}	5.61±0.10^{a}	5.13±0.16^{a}	1.01±0.39^{a}	1.12±0.08^{a}	0.34±0.00^{a}	0.38±0.03^{b}
17	YARW_B	24.67±2.12^{a}	36.86±1.08^{b}	10.57±0.43^{a}	8.98±0.44^{b}	2.75±0.10^{a}	2.23±0.22^{b}	0.16±0.05^{a}	0.17±0.01^{a}
18	YARW_W	36.53±4.08^{a}	46.28±1.44^{b}	5.14±0.13^{a}	4.98±0.12^{a}	1.34±0.17^{a}	1.16±0.38^{a}	0.29±0.01^{a}	0.33±0.02^{b}
19	KWA_B	26.28±1.11^{a}	35.69±0.53^{b}	11.01±0.20^{a}	9.77±0.20^{b}	2.32±0.08^{a}	2.07±0.10^{a}	0.17±0.00^{a}	0.18±0.01^{a}
20	KWA_W	36.79±0.36^{a}	45.83±0.76^{b}	5.18±0.09^{a}	5.06±0.19^{a}	0.88±0.35^{a}	1.08±0.00^{a}	0.27±0.00^{a}	0.39±0.04^{b}
21	YARK_B	25.23±2.25^{a}	37.47±2.89^{b}	8.09±0.12^{a}	7.72±0.41^{a}	3.23±0.17^{a}	2.20±0.20^{b}	0.16±0.00^{a}	0.18±0.01^{a}
22	YARK_W	37.43±2.25^{a}	45.60±1.04^{b}	5.29±0.19^{a}	5.16±0.15^{a}	1.35±0.58^{a}	1.07±0.10^{a}	0.32±0.01^{a}	0.36±0.09^{b}
23	DANB_B	24.33±4.16^{a}	35.33±3.79^{b}	10.02±0.83^{a}	9.11±1.34^{b}	2.72±0.55^{a}	3.38±0.60^{b}	0.15±0.00^{a}	0.16±0.00^{a}
24	DANB_W	54.00±17.78^{a}	46.00±2.00^{b}	4.67±0.73^{a}	4.79±0.23^{a}	0.90±0.39^{a}	1.03±0.34^{a}	0.33±0.00^{a}	0.38±0.06^{b}
25	BAD_B	25.33±3.21^{a}	28.00±3.61^{a}	9.80±0.90^{a}	9.09±1.00^{b}	3.50±0.57^{a}	3.06±0.70^{b}	0.15±0.00^{a}	0.16±0.00^{a}
26	BAD_W	36.00±1.73^{a}	65.33±17.79^{b}	4.55±0.72^{a}	4.79±0.72^{a}	1.13±0.39^{a}	0.78±0.25^{b}	0.36±0.06^{a}	0.36±0.03^{a}
27	FARO_B	25.67±7.23^{a}	27.63±3.51^{a}	9.62±1.33^{a}	9.00±1.67^{b}	2.49±0.75^{a}	2.03±0.00^{b}	0.15±0.00^{a}	0.16±0.01^{a}
28	FARO_W	44.33±2.65^{a}	43.67±3.21^{a}	4.56±0.27^{a}	4.81±0.22^{a}	0.90±0.39^{a}	1.13±0.39^{a}	0.29±0.00^{a}	0.36±0.03^{b}
29	JIR_B	33.00±5.20^{a}	35.67±6.66^{a}	10.39±0.84^{a}	8.67±0.63^{b}	2.59±0.40^{a}	2.80±0.21^{a}	0.19±0.00^{a}	0.18±0.01^{a}
30	JIR_W	54.00±17.35^{a}	46.33±14.57^{b}	4.36±1.11^{a}	4.47±0.18^{a}	1.03±0.00^{a}	1.16±0.17^{a}	0.33±0.07^{a}	0.37±0.06^{b}
31	YARM_B	34.00±5.29^{a}	36.33±2.52^{a}	7.81±0.22^{a}	8.23±0.59^{a}	3.15±0.41^{a}	2.80±0.22^{b}	0.18±0.02^{a}	0.15±0.01^{b}
32	YARM_W	48.33±15.31^{a}	51.67±17.01^{a}	4.57±0.50^{a}	4.79±0.69^{a}	1.01±0.58^{a}	0.90±0.39^{a}	0.34±0.05^{a}	0.35±0.08^{a}

Mean ±SD values between male and female denoted by the same letters in superscript are not significantly different ($p > 0.05$) using two-way ANOVA, multiple comparison was done using *Bonferroni's* test. Brown rice (_B), White rice (_W), (n=30). Triglyceride (TG), Superoxide dismutase (SOD), Catalase (CAT), Malondialdehyde (MDA). NCD= Normal diet, HFD= High fat diet, *Akai maza hajj* =AKM, *Baingila* =BAI, *Baburashi* =BAB, *Jamila* =JAM, *Maibakincarki* =MBC, *Yarwasagi* =YARW, *Kwandala* =KWA, *Yarkatabore* =YARK, *Danboto* =DANB, *Bakindanboto* =BAD, *Faro 44* =FARO, *Jirkita* =JIR, *Yarkukuma* =YARM, *Dankaushi* =DAN and *Jeep* =JEEP.

Table S4. *dIRS*, *dPEPCK*, and *dACC* expressions in male and female flies exposed to different rice cultivars.

		dIRS FOLD CHANGE		*dPEPCK* FOLD CHANGE		*dACC* FOLD CHANGE	
		**MALE**	**FEMALE**	**MALE**	**FEMALE**	**MALE**	**FEMALE**
1	HFD	1.78±0.13^{a}	0.59±0.00^{b}	4.04±0.16^{a}	2.15±0.09^{b}	8.40±0.51^{a}	5.09±0.57^{b}
2	AKM_B	4.63±1.76^{a}	1.93±0.19^{b}	2.20±0.11^{a}	0.89±0.08^{b}	2.71±0.40^{a}	2.12±0.23^{a}
3	AKM_W	1.62±0.13^{a}	0.66±0.03^{b}	14.63±2.57^{a}	2.09±0.44^{b}	17.86±1.66^{a}	5.04±0.49^{b}
4	BAI_B	4.09±0.47^{a}	1.63±0.59^{b}	2.18±0.50^{a}	1.01±0.04^{b}	3.14±0.35^{a}	2.04±0.50^{b}
5	BAI_W	1.43±0.57^{a}	0.76±0.16^{b}	4.60±0.12^{a}	5.64±0.18^{b}	7.94±2.35^{a}	4.44±1.31^{b}
6	BAB_B	4.84±0.14^{a}	2.07±0.52^{b}	1.86±0.37^{a}	0.76±0.09^{b}	2.68±0.14^{a}	1.75±0.07^{b}
7	BAB_W	1.41±0.66^{a}	0.77±0.93^{b}	4.13±0.88^{a}	2.23±0.25^{b}	9.29±0.52^{a}	5.47±0.78^{b}
8	DAN_B	5.07±0.84^{a}	1.55±0.17^{b}	2.19±0.11^{a}	0.79±0.43^{b}	3.41±0.95^{a}	1.50±0.13^{b}
9	DAN_W	1.35±0.19^{a}	0.86±0.23^{b}	5.10±1.19^{a}	6.08±0.47^{b}	9.79±0.84^{a}	4.52±0.40^{b}
10	JEEP_B	4.53±0.34^{a}	2.04±0.02^{b}	2.15±0.21^{a}	1.00±0.02^{b}	2.94±0.29^{a}	1.93±0.28^{b}
11	JEEP_W	1.36±0.42^{a}	0.84±0.06^{b}	4.37±0.59^{a}	2.98±0.70^{b}	8.68±0.76^{a}	5.15±0.44^{b}
12	JAM_B	4.35±0.21^{a}	2.05±0.33^{b}	2.03±0.57^{a}	1.06±0.07^{b}	3.00±0.05^{a}	1.50±0.00^{b}
13	JAM_W	1.50±0.27^{a}	0.70±1.41^{b}	4.55±1.63^{a}	3.04±0.14^{b}	7.50±0.42^{a}	5.40±0.14^{b}
14	MBC_B	4.40±0.38^{a}	2.02±0.40^{b}	1.95±0.64^{a}	1.07±0.08^{b}	2.50±0.57^{a}	1.75±0.21^{b}
15	MBC_W	1.45±0.14^{a}	0.55±0.34^{b}	5.10±0.85^{a}	2.45±0.35^{b}	7.95±2.33^{a}	4.85±0.78^{b}
16	YARW_B	4.40±1.69^{a}	1.75±0.02^{b}	2.15±0.35^{a}	1.05±0.06^{b}	3.05±0.21^{a}	1.70±0.08^{b}
17	YARW_W	1.35±0.05^{a}	0.60±0.14^{b}	4.15±0.92^{a}	2.30±0.57^{b}	8.95±0.35^{a}	5.25±0.20^{b}
18	KWA_B	4.55±1.04^{a}	2.05±0.05^{b}	2.18±0.14^{a}	0.80±0.28^{b}	2.85±0.49^{a}	1.85±0.49^{b}
19	KWA_W	1.35±0.03^{a}	0.54±0.09^{b}	6.05±0.07^{a}	3.05±0.35^{b}	9.20±2.69^{a}	4.40±0.57^{b}
20	YARK_B	4.30±0.55^{a}	1.95±0.18^{b}	1.95±0.64^{a}	0.85±0.07^{b}	2.52±0.64^{a}	1.60±0.05^{b}
21	YARK_W	1.30±0.02^{a}	0.85±0.01^{b}	5.04±1.27^{a}	4.55±1.48^{a}	9.65±0.78^{a}	4.65±0.49^{b}
22	DANB_B	4.16±0.33^{a}	2.02±0.21^{b}	2.01±0.14^{a}	0.80±0.34^{b}	2.99±0.08^{a}	2.02±0.27^{b}
23	DANB_W	1.48±0.04^{a}	0.81±0.01^{b}	4.02±0.61^{a}	2.25±0.90^{b}	7.50±0.30^{a}	4.42±0.04^{b}
24	BAD_B	4.14±1.12^{a}	2.01±0.82^{b}	2.06±0.04^{a}	0.75±0.06^{b}	2.64±0.41^{a}	1.99±0.15^{b}
25	BAD_W	1.79±0.15^{a}	0.09±0.00^{b}	4.60±0.57^{a}	3.04±0.17^{b}	7.59±1.46^{a}	4.67±0.88^{b}
26	FARO_B	4.78±0.31^{a}	1.92±0.33^{b}	1.91±0.02^{a}	0.72±0.04^{b}	2.97±0.05^{a}	1.88±0.04^{b}
27	FARO_W	1.62±0.09^{a}	0.12±0.04^{b}	4.60±0.13^{a}	2.67±0.08^{b}	8.19±1.00^{a}	4.76±0.43^{b}
28	JIR_B	4.69±0.76^{a}	2.05±0.23^{b}	1.87±0.28^{a}	0.84±0.08^{b}	2.32±0.61^{a}	2.32±0.15^{a}
29	JIR_W	1.70±0.16^{a}	0.13±0.07^{b}	4.24±0.39^{a}	2.29±0.39^{b}	8.02±0.02^{a}	4.09±0.76^{b}
30	YARM_B	4.63±0.05^{a}	1.82±0.18^{b}	1.78±0.14^{a}	0.80±0.06^{b}	2.80±0.01^{a}	2.19±0.29^{a}
31	YARM_W	1.88±0.10^{a}	0.73±0.06^{b}	4.40±0.83^{a}	2.62±0.08^{b}	7.71±1.00^{a}	4.40±0.15^{b}

Fold change was calculated using Livak method ($2^{-\Delta\Delta CT}$), Mean ±SD values between male and female denoted by the same letters in superscript are not significantly different ($p > 0.05$) using two-way ANOVA, multiple comparison was done using *Bonferroni's* test. (n=20), _B= Brown rice, _W= White rice. HFD= High fat diet, *Akai maza hajj* =AKM, *Baingila* =BAI, *Baburashi* =BAB, *Jamila* =JAM, *Maibak-incarki* =MBC, *Yarwasagi* =YARW, *Kwandala* =KWA, *Yarkatabore* =YARK, *Danboto* =DANB, *Bak-indanboto* =BAD, *Faro 44* =FARO, *Jirkita* =JIR, *Yarkukuma* =YARM, *Dankaushi* =DAN and *Jeep* =JEEP.
